# Epidemiological investigation of porcine circovirus type 2 and its coinfection rate in Shandong province in China from 2015 to 2018

**DOI:** 10.1186/s12917-020-02718-4

**Published:** 2021-01-07

**Authors:** Zicheng Ma, Mengda Liu, Zhaohu Liu, Fanliang Meng, Hongyu Wang, Longlong Cao, Yan Li, Qiulin Jiao, Zifeng Han, Sidang Liu

**Affiliations:** 1 College of Animal Science and Veterinary Medicine, Shandong Agricultural University, 271018 Taian, China; 2 Laboratory of Zoonoses, Animal Health and Epidemiology Center, 266032 Qingdao, China; 3 Emergency Centre for the Control of Transboundary Animal Diseases, Food and Agriculture Organization of the United Nations (FAO), 100600 Beijing, China

**Keywords:** Porcine circovirus, Veterinary epidemiology, Coinfection, Phylogenetic analysis, Shandong province

## Abstract

**Background:**

Porcine circovirus type 2 (PCV2) is one of the crucial swine viral pathogens, caused porcine circovirus associated diseases (PCVAD). Shandong province is one of the most important pork producing areas and bears a considerable economic loss due to PCVAD. However, there is limited information on epidemiology and coinfection rate of PCV2 with other critical swine diseases in this area, such as porcine reproductive and respiratory syndrome virus (PRRSV), classical swine fever virus (CSFV), Pseudorabies virus (PRV), and porcine epidemic diarrhea virus (PEDV).

**Results:**

Overall, 89.59% serum samples and 36.98% tissue samples were positive for PCV2 specified ELISA and PCR positive for PCV2, respectively. The coinfection rates of PCV2 with PRRSV, PRV, CSFV, and PEDV were 26.73%, 18.37%, 13.06%, and 3.47%, respectively. Moreover, genetic characteristic of PCV2 were analyzed based on the cap genes showing that PCV2d is the dominant sub-genotype circulating in the province.

**Conclusions:**

Our findings reveal that PCV2d, as the dominant strain, is prevailing in pig farms in Shandong province at high levels. There was a high frequency of coinfection of PCV2 and PRRSV.

## Background

Porcine Circoviruses (PCVs) are single-standard circular DNA virus, which belong to the member of genus *Circovirus* within the family of *Circoviridae* [1]. PCVs includes PCV1, PCV2, and PCV3. PCV1 was initially found in a contaminant of porcine kidney 15 cells in 1974, known as non-pathogenic [2]. Unlike PCV1, PCV2 can cause various clinical signs, such as postweaning multisystemic wasting syndrome (PMWS), porcine dermatitis and nephropathy syndrome (PDNS), enteritic disease, and reproductive failure in breeding pigs [3]. PCV2 has been recognized as an economically important swine pathogen that threaten the global swine industry.

As one of the smallest animal viruses, PCV2 has a closed single-stranded DNA genome of 1766–1769 nucleotides with no envelope [4]. The genome contains 11 open reading frames (ORFs), of which four ORFs were well-characterized [5, 6]. PCV2 ORF1 and ORF2 encode replicase (Rep) and capsid (Cap) protein that are of great importance for virus entry and its replication [7, 8]. It was reported that ORF2 gene shows more genetic variations compared to ORF1, which is commonly used as a phylogenetic and epidemiological marker [9]. The evolution of PCV2 was resulted from genetic mutations and combination, which leads to the generation of genetic diversity. Genotypic shifts may be relevant to changes in pathogenicity and vaccine immunity. The European Consortium on PCV disease proposed pairwise sequence comparisons and linearized phylogenetic trees in 2008, which classified PCV2 strains into three genotypes, including PCV2a, PCV2b, and PCV2c [10]. The PCV2d genotype was firstly report in 2010 [11], and it has been identified as a predominant genotype in numerous countries [12–16].

Co-infection of viruses plays a critically important role in animal disease management and control in the field [14, 17]. Besides of PCVs, there are other viral pathogens threatening the pig raising industry at regional, national and global level, including porcine epidemic diarrhea virus (PEDV), porcine reproductive and respiratory syndrome virus (PRRSV), pseudorabies virus (PRV), classical swine fever virus (CSFV). Previous investigation has shown that PCV2 pathogenesis can be exacerbated by a coinfection with an additional swine viral disease, such as PRRSV and PEDV [18, 19].

Shandong province, as one of the most essential regions in China, which raises dense pig population. It was estimated that there were 28 million pigs and 3 million sows produced in Shandong province in 2017. However, there is lack of information on PCV2 prevalence and its coinfection with other crucial swine viral diseases in intensive pig farms in Shandong. The goal of this study was to elucidate the epidemiological and evolutionary dynamics of PCV2, as well as its coinfection rates in Shandong province from 2015 to 2018.

## Results

### Seroprevalence Rate (SPR) of PCV2

Serum PCV2 antibodies were tested by ELISA in different herds from 2015 to 2018. Consistent with previous studies in other provinces of China [20, 21], the overall SPR of PCV2 was shown as approximately 90% in the studied pig farms in the province, as described in Table 1. SPR of PCV2 was gradually increased from the bottom year in 2015 (87.27%) to the highest level (92.43%) in 2018. Moreover, the lowest average S/P ratio (1.01) was found in young piglets ranging from 0.91 to 1.03. The average S/P ratio of fattening pigs was 1.55 with the highest (1.81) in 2016 and the lowest (1.43) in 2018. The remaining three herds showed an averaging S/P ratio at approximately 1.72.

**Table 1:** Seroprevalence rate of PCV2 and S/P ratios in different herds in Shandong Province from 2015 to 2018

Year	Number of samples	Number of positive samples	SPR	Different Herds				
				Sows	Replacement pigs	Boars	Young piglets (< 100 d)	Fattening pigs (> 100 d)
2015	1257	1097	87.27%	1.78 ± 0.04	1.79 ± 0.03	1.48 ± 0.01	0.97 ± 0.02	1.51 ± 0.07
2016	1850	1642	88.76%	1.80 ± 0.02	1.43 ± 0.02	1.66 ± 0.02	1.01 ± 0.01	1.81 ± 0.05
2017	2121	1895	89.34%	1.74 ± 0.02	1.61 ± 0.02	1.72 ± 0.03	1.03 ± 0.02	1.66 ± 0.04
2018	1758	1625	92.43%	1.56 ± 0.01	1.47 ± 0.03	1.41 ± 0.01	0.91 ± 0.03	1.43 ± 0.03
Total	6986	6259	89.59%	1.77 ± 0.01	1.70 ± 0.02	1.68 ± 0.01	1.01 ± 0.02	1.55 ± 0.03

### PCR positive rate of PCV2

All 1,325 tissue samples from Shandong province were tested by PCR, of which 490 samples were shown as positive of PCV2. The results of positive rate of PCV2 were shown in different years (Table 2). The overall average positive rate of PCV2 reached 36.98% during the investigation period, with the highest rate (43.21%) observed in 2017 (191/442) and lowest (31.16%) in 2016 (91/292).

**Table 2:** The positive rate of PCV2 DNA from 2015 to 2018

Year	Number of samples	Number of positive samples	Positive rate
2015	140	56	40.00%
2016	292	91	31.16%
2017	442	191	43.21%
2018	451	152	33.70%
Total	1325	490	36.98%

### Phylogenetic analysis of PCV2

**Fig. 1:**
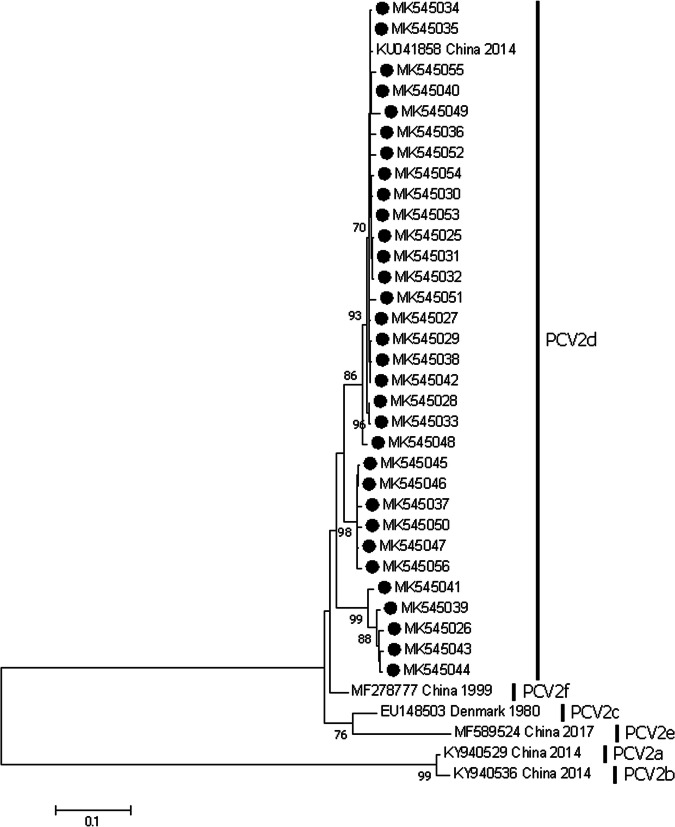
The phylogenetic analysis based on the cap genes of PCV2. Phylogenetic trees were constructed with the MEGA 7.0.14 software using the neighbor-joining (NJ) method, with the Jukes-cantor model as a nucleotide substitution model. The reliability of the generated trees was determined with 1000 replicates of the data set. Thirty-two isolates and six reference sequences were analyzed

Recent studies have indicated a new genotype shifting from PCV2b to PCV2d in various regions in China [15, 22–24], indicating that PCV2d has become a dominant genotype. In order to understand current dominant strain and genotype in this field, a phylogenetic tree was developed based on ORF2 sequences of 32 PCV2 gained in 2018 and 18 ORF2 from PCV2 reference strains deposited in GenBank database. Table 3 summarizes the geographic distribution, genotype, tissue, and GenBank accession number of the investigated PCV2 strains. Interestingly, all 32 ORF2 sequences were clustered with PCV2d isolates (Fig. 1).

**Table 3:** Information of positive samples and GenBank accession number of PCV2-Cap genes

No.	City	Genotype	Tissue	Genbank accession number
1	Liaocheng	PCV2d	lymph	MK545025
2	Tai’an	PCV2d	lymph	MK545026
3	Qingdao	PCV2d	lymph	MK545027
4	Laiwu	PCV2d	lymph	MK545028
5	Dongying	PCV2d	lymph	MK545029
6	Tai’an	PCV2d	lymph	MK545030
7	Binzhou	PCV2d	lymph	MK545031
	Yantai	PCV2d	spleen	
	Ji’nan	PCV2d	lymph	
	Rizhao	PCV2d	spleen	
	Ji’nan	PCV2d	lymph/spleen	
8	Jining	PCV2d	lymph	MK545032
9	Dongying	PCV2d	lymph	MK545033
10	Rizhao	PCV2d	lymph	MK545034
11	Ji’nan	PCV2d	lymph	MK545035
	Weifang	PCV2d	lymph	
	Ji’nan	PCV2d	lymph/spleen	
12	Zaozhuang	PCV2d	lymph	MK545036
13	Jinan	PCV2d	lymph	MK545037
14	Tai’an	PCV2d	spleen	MK545038
15	Liaocheng	PCV2d	spleen	MK545039
16	Liaocheng	PCV2d	lymph/spleen	MK545040
17	Heze	PCV2d	lymph	MK545041
18	Tai’an	PCV2d	lymph	MK545042
	Heze	PCV2d	lymph	
19	Ji’nan	PCV2d	lymph	MK545043
20	Dongying	PCV2d	lymph	MK545044
21	Weifang	PCV2d	lymph	MK545045
22	Ji’nan	PCV2d	lung	MK545046
23	Liaocheng	PCV2d	lymph	MK545047
24	Tai’an	PCV2d	lymph	MK545048
25	Binzhou	PCV2d	lymph/spleen	MK545049
26	Weihai	PCV2d	lymph	MK545050
27	Liaocheng	PCV2d	lymph	MK545051
28	Zaozhuang	PCV2d	spleen	MK545052
29	Heze	PCV2d	lymph	MK545053
30	Tai’an	PCV2d	lymph	MK545054
31	Liaocheng	PCV2d	lymph/spleen	MK545055
32	Liaocheng	PCV2d	lymph	MK545056

### Co-infection rate

All PCV2-PCR positive samples were measured for the co-infection status of PRRSV, CSFV, PRV and PEDV. They were demonstrated depending on areas (Table 4). In our study, 26.73% of PCV2 positive pigs were found to be co-infected by PRRSV, followed with 18.37% (90/490) by PRV. The coinfection by PCV2 and CSFV was 13.06% (64/490). The positive rate of PCV2 and PEDV coinfection was 3.47% (17/490). The co-infection rates of PRV and PCV2 were over 18.37% (90/490) with the highest in Tai’an (28/90), followed by Linyi (15/90) Ji’ning (11/90), and Liaocheng (10/90).

**Table 4:** Positive rate of PCV2 and its coinfection rate in different cities in Shandong Province from 2015–2018

City	Number of positive samples	Positive rate	PCV2 + PRRSV	PCV2 + PRV	PCV2 + CSFV	PCV2 + PEDV
Linyi	89	18.16%	28	15	14	2
Tai’an	61	12.45%	19	28	3	1
Ji’ning	44	8.98%	15	11	6	2
Liaocheng	41	8.37%	7	10	9	0
Laiwu	40	8.16%	15	5	7	3
Dongying	36	7.35%	8	4	1	0
Binzhou	34	6.94%	6	3	5	1
Ji’nan	29	5.92%	9	1	3	1
Weifang	29	5.92%	9	2	3	0
Heze	26	5.31%	3	1	8	3
Zibo	16	3.27%	6	3	1	2
Dezhou	12	2.45%	2	2	1	0
Rizhao	10	2.04%	1	1	2	0
Weihai	7	1.43%	2	0	0	1
Yantai	7	1.43%	0	0	1	0
Zaozhuang	5	1.02%	1	3	0	1
Qingdao	4	0.82%	0	1	0	0
**Total**	490		131/490 (26.73%)	90/490 (18.37%)	64/490 (13.06%)	17/490 (3.47%)

### Sequence alignment of cap protein

**Fig. 2:**
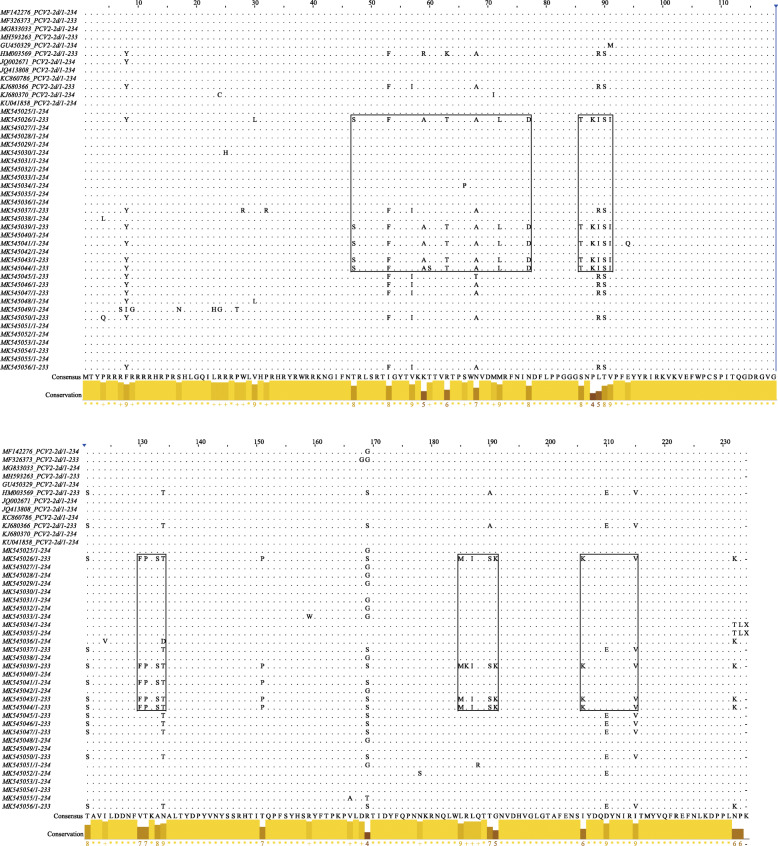
Amino acid sequence alignment of cap protein of 32 PCV2 isolates. The 32 isolates and 12 reference cap protein were aligned by clustal W method in MEGA6.0 software

The multiple alignments of PCV2 Cap protein were carried out by a Clustal W method to evaluate the amino acid mutations of Cap protein. Consistent with previous studies [11], typical motif 86TNKISI91 was found in result of the PCV2 Cap alignment analysis. Moreover, other typical motifs 129FFPKST134 and 185MRIQTSK192 were observed in PCV2d strains. In addition, other amino acid substitutions at different sites were summarized in Fig. 2.

## Discussion

Since 1990s, PCV2 has been considering as one of the essential viral pathogen of pigs globally [25, 26], which caused great economic loss in the swine industry. There is a high prevalence observed in numerous swine-farming countries. Previous investigations have indicated that the positive rate of PCV2 in swine farms in China was over 50% [15, 20, 27]. As one of the most vital regions in China regarding breeding food animals, Shandong province raises dense pig population, which suffered PCV2 in recent years. Our study investigated PCV2 seroprevalence, genetic characterization, and its confection rates with other essential swine viral diseases in the field. Results of this study provide useful information on tackling the spread of PCV2 in the province.

Depending on the phylogenetic analysis of cap genes, PCV2 can be divided into five genotypes, defined as PCV2a, PCV2b, PCV2c, PCV2d, and PCV2e [28–30]. Previous studies indicated that PCV2a and PCV2b were the two major genotypes of PCV2 circulating in China with positive rates exceeding 50% from 2006 to 2007 [20, 31]. Since 2008, while there was an observed decrease of positive rate of PCV2b, positive rates of PCV2d showed an increase up to approximately 50% were found in samples collected in the field, indicating that PCV2d became the predominant genotype after 2008 [18, 24]. PCV2e contains an additional five amino acid at the C-terminal end resulting in 238 amino acids of ORF2, which has been recently identified as a new genotype [31]. In China, with an exception of PCV2c which was only reported in Denmark [32], other four genotypes are circulating in the field. A genotype shifting from PCV2a and PCV2b to PCV2d in many countries since 2012 [14, 15, 18]. In the present study, PCV2d as a dominant the genotype was highly prevalent in the field in Shandong province in China. Furthermore, several specific motifs were identified, 86 TNKISI91, 129FFPKST134 and 185MRIQTSK192 in PCV2d strains. Additionally, some of the amino acid mutations identified in this study are associated with antibody recognitions and virulence of the PCV2, which may be relevant to the immune escape mechanisms [33].

It is commonly for pigs to be identified as coinfection with different pathogens in the field conditions [14, 17, 34]. Studies in the past demonstrated that pigs can be coinfected by PCV2 with numerous pathogens, such as PRRSV, CSFV, PRV, and PEDV, etc. Previous investigations reported that there was approximately 21.9–52.3% of coinfection rates of PRRSV and PCV2 [11, 34–36]. The coinfection rate of PCV2 and PRV was shown as 35% in field samples [37]. Approximately 50% and 80% samples collected at sample and farm level were shown as PCV2 and PEDV coinfection [14]. In this study, different patterns of combined infections were tested with the positive rates ranged from 26.73% (PCV2 + PRRSV) to 3.47% (PCV2 + PEDV) (Table 4). Our results indicate that there is a high frequency of coinfection of PCV2 and PRRSV in Shandong province. Combined with previous investigations, results in this study indicated that different patterns of dual infections are often seen in intensive swine breeding system in China. Moreover, further discussions on the refinement of animal disease control strategies was driven by the complicated co-infection status shown in the results [14, 17, 20, 34–36]. It is therefore of overriding importance to take forceful measures, such as strengthening biosecurity, monitoring and surveillance, and improving environmental hygiene.

## Conclusions

We investigated the molecular characterization and prevalence of PCV2 as well as its co-infection statues in Shandong province from 2015 to 2018. Our results clearly reveal that PCV2d, as the dominant strain, is prevailing at high levels in intensive pig farms in the investigated province. Furthermore, we found new mutations which may be related to antibody recognitions and virulence of PCV2 and immune escape mechanisms. It also provided data and insights on co-infection status of PCV2 and other major swine viral diseases, which can improve the understanding from epidemiological perspective and may contribute the prevention and control swine diseases in China.

## Methods

### Experimental Protocols

Serum samples were collected from 277 intensive pig farms (≥ 300 sows) that located in 17 cities in Shandong Province. They were categorized as following groups, including sow, replacement pig, boar, fatting pig, and piglets. By using PCR or RT-PCR, tissue samples were randomly selected from local slaughterhouses to test positive rate of PCV2 and its coinfection rate with PRRSV, PRV, CSFV, and PEDV, respectively.

### Serum sample collection and ELISA

A total number of 6,986 serum samples were collected from 17 cities in Shandong, covering the entire province. Blood collection through the jugular vein and the animal were released. Commercial ELISA kits (MEDIAN Diagnostics, Korea) were used according to the manufacturer’s instructions to differentiate the vaccine strain from field strains. The serum sample was diluted 1:100 times, and the detection process was carried out according to the instructions. After the operation, the sample plate was placed at the wavelength of 450 nm to measure the OD value of each hole. When the OD value of positive control ≥ 0.5 and the OD value of negative control ≤ 0.3, the experiment is established. Standards for negative and positive samples: S/P value ≥ 0.4 is considered positive; S/P values < 0.3 were considered negative; 0.3 ≤ S/P values < 0.4 were considered suspicious. SP = (sample value-negative value) / (positive value-negative value).

### Primer design and virus detection

One thousand three hundred twenty-five tissue samples, including lymph gland, spleen, and brain samples were collected from several slaughterhouses in Shandong province. All pigs bled to death after electronarcosis. According to the manufacturer’s instructions, commercial kits (Takara Biomedical Technology, Beijing, China) were used to extract viral DNA or RNA of the samples. All primers used in this study were summarized in Table 5. Extracted DNA was used as template to detect PRV and PCV2 by PCR. RNA was used to detect PEDV, CSFV and PRRSV by RT-PCR.

The PCR cycle profile and cycling conditions have been described previously [34]. All sequence of primers used and relevant X values refer to annealing temperature are summarized in Table 5. PCR products were subjected to electrophoresis on 1% agarose gels stained with ethidium bromide and visualized with an ultraviolet light transilluminator. The PCR product is recovered by gel, connected to the pMD 18-T vector (Takara Biomedical Technology, Beijing, China), transformed into DH5α competent cells, and cultured for enrichment. The bacterial liquid identified as positive for the gE gene was sent to Company (Sangon Biotech, Shanghai, China) for sequencing.

**Table 5:** All primers used in this study

Primer name	Sequences	Annealing Temperature (℃)	Product size(bp)
PCV2-F	CCATATGAAATAAATTACTGAG	51	785
PCV2-R	CAGCGCACTTCTTTCGTTTTCAG		
CSFV-F	TRACCAYGCAYATGWCAGAAGTACC	57	599
CSFV-R	AGTCGACTTCCCTGGTTTCACTTG		
PRRSV-F	GCGGATCCATGCCAAATAACAAC	55	372
PRRSV-R	AGCTCGAGTCATGCTGAGGGTGA		
PRV-F	TCCACTCGCAGCTCTTCT	57	632
PRV-R	GCACGTCATCACGAAGGA		
PEDV-F	TTCCCGTTGATGAGGTGAT	51	552
PEDV-R	AAGCATTGACTGAACGACC		

### Phylogenetic analysis

The Multiple sequence alignments were conducted by Clustal W of the Megalign program (DNAStar software/Sequence sorting), MEGA 7 (mega software/linearized phylogenetic trees), Bioedit (Bioedit software/genetics analysis). 17 reference PCV2 genome sequences were obtained from the GenBank database to compare and determine the genotype of the PCV2 isolates.

## Data Availability

The datasets generated and analysed during the current study are available in the NCBI repository. **32 isolates sequences**: MK545025: https://www.ncbi.nlm.nih.gov/nuccore/MK545025. MK545026: https://www.ncbi.nlm.nih.gov/nuccore/MK545026. MK545027: https://www.ncbi.nlm.nih.gov/nuccore/MK545027. MK545028: https://www.ncbi.nlm.nih.gov/nuccore/MK545028. MK545029: https://www.ncbi.nlm.nih.gov/nuccore/MK545029. MK545030: https://www.ncbi.nlm.nih.gov/nuccore/MK545030. MK545031: https://www.ncbi.nlm.nih.gov/nuccore/MK545031. MK545032: https://www.ncbi.nlm.nih.gov/nuccore/MK545032. MK545033: https://www.ncbi.nlm.nih.gov/nuccore/MK545033. MK545034: https://www.ncbi.nlm.nih.gov/nuccore/MK545034. MK545035: https://www.ncbi.nlm.nih.gov/nuccore/MK545035. MK545036: https://www.ncbi.nlm.nih.gov/nuccore/MK545036. MK545037: https://www.ncbi.nlm.nih.gov/nuccore/MK545037. MK545038: https://www.ncbi.nlm.nih.gov/nuccore/MK545038. MK545039: https://www.ncbi.nlm.nih.gov/nuccore/MK545039. MK545040: https://www.ncbi.nlm.nih.gov/nuccore/MK545040. MK545041: https://www.ncbi.nlm.nih.gov/nuccore/MK545041. MK545042: https://www.ncbi.nlm.nih.gov/nuccore/MK545042. MK545043: https://www.ncbi.nlm.nih.gov/nuccore/MK545043. MK545044: https://www.ncbi.nlm.nih.gov/nuccore/MK545044. MK545045: https://www.ncbi.nlm.nih.gov/nuccore/MK545045. MK545046: https://www.ncbi.nlm.nih.gov/nuccore/MK545046. MK545047: https://www.ncbi.nlm.nih.gov/nuccore/MK545047. MK545048: https://www.ncbi.nlm.nih.gov/nuccore/MK545048. MK545049: https://www.ncbi.nlm.nih.gov/nuccore/MK545049. MK545050: https://www.ncbi.nlm.nih.gov/nuccore/MK545050. MK545051: https://www.ncbi.nlm.nih.gov/nuccore/MK545051. MK545052: https://www.ncbi.nlm.nih.gov/nuccore/MK545052. MK545053: https://www.ncbi.nlm.nih.gov/nuccore/MK545053. MK545054: https://www.ncbi.nlm.nih.gov/nuccore/MK545054. MK545055: https://www.ncbi.nlm.nih.gov/nuccore/MK545055. MK545056: https://www.ncbi.nlm.nih.gov/nuccore/MK545056. **17 reference sequences**: KU041858: https://www.ncbi.nlm.nih.gov/nuccore/KU041858. MF278777: https://www.ncbi.nlm.nih.gov/nuccore/MF278777. EU148503: https://www.ncbi.nlm.nih.gov/nuccore/EU148503. MF589524: https://www.ncbi.nlm.nih.gov/nuccore/MF589524. KY940529: https://www.ncbi.nlm.nih.gov/nuccore/KY940529. KY940536: https://www.ncbi.nlm.nih.gov/nuccore/KY940536. MF142276: https://www.ncbi.nlm.nih.gov/nuccore/MF142276. MF326373: https://www.ncbi.nlm.nih.gov/nuccore/MF326373. MG833033: https://www.ncbi.nlm.nih.gov/nuccore/MG833033. MH593263: https://www.ncbi.nlm.nih.gov/nuccore/MH593263. GU450329: https://www.ncbi.nlm.nih.gov/nuccore/GU450329. HM003569: https://www.ncbi.nlm.nih.gov/nuccore/HM003569. JQ002671: https://www.ncbi.nlm.nih.gov/nuccore/JQ002671. JQ413808: https://www.ncbi.nlm.nih.gov/nuccore/JQ413808. KC860786: https://www.ncbi.nlm.nih.gov/nuccore/KC860786. KJ680366: https://www.ncbi.nlm.nih.gov/nuccore/KJ680366. KJ680370: https://www.ncbi.nlm.nih.gov/nuccore/KJ680370.

## Abbreviations

CSV: Classical swine fever virus
PCV2: Porcine circovirus type 2
PEDV: Porcine epidemic diarrhea virus
PRRSV: Porcine reproductive and respiratory syndrome virus
PRV: Pseudorabies virus
SPR: Seroprevalence rate
